# Polarimetric Metagrating‐Assisted One‐Step Multi‐Dimensional Sensing Using a Vector Vortex Beam

**DOI:** 10.1002/nap2.70263

**Published:** 2026-08-24

**Authors:** Han Gao, Zhelei Chen, Kaihao Deng, MinJun Xie, Huanxin Liao, Zeyu Zheng, Hao Yang, ChunLian Zhan, Yanlong Meng, Bo Xiong, Wei Ma

**Affiliations:** ^1^ China Jiliang University College of Optical and Electronic Technology Hangzhou China; ^2^ Zhejiang Province Key Laboratory of Optoelectronic Metrology and Precision Instruments Hangzhou China; ^3^ College of Information Science and Electronic Engineering Zhejiang University Hangzhou China

## Abstract

Multidimensional optical sensing requires compact methods capable for jointly retrieving spatial, angular, and polarization‐resolved information from structured optical fields. Here, we present a one‐step multidimensional sensing approach in which vector vortex beams (VVBs) serve as optical probes, whereas a polarimetric metagrating simultaneously analyzes their intensity and polarization responses to radial and angular variations. Theoretical and experimental investigations of the topological charge *l* and polarization order *m* reveal their distinct governing roles, establishing a parameter‐space framework that links intrinsic VVB properties to measurable radial and polarization‐dependent angular features. The polarimetric diffraction orders reconstruct the full‐Stokes parameters and reveal a periodic angle‐of‐polarization distribution governed by *2m*, whereas the polarization‐insensitive central order preserves the VVB intensity morphology and yields a dark‐core radius with a square‐root dependence on *l*. Measurements with linearly and elliptically polarized VVBs demonstrate an experimentally distinguishable angular change of approximately 2°. Using the polarization‐insensitive central diffraction order for radius sensing extends the metagrating beyond polarization analysis and broadens its functionality in diverse polarimetric scenarios. The integration of the topological and polarization degrees of freedom of VVBs with chip‐scale full‐Stokes polarimetry establishes a compact and robust platform for vector‐beam diagnostics, structured‐light state recognition, optical mode monitoring, and polarization‐dependent rotation sensing.

## Graphical abstract

One‐step multidimensional sensing is realized by combining VVB probes with a polarimetric metagrating, where the full‐Stokes diffraction orders retrieve *m*‐dependent angular features and the polarization‐insensitive central order extracts *l*‐dependent radial‐intensity information.

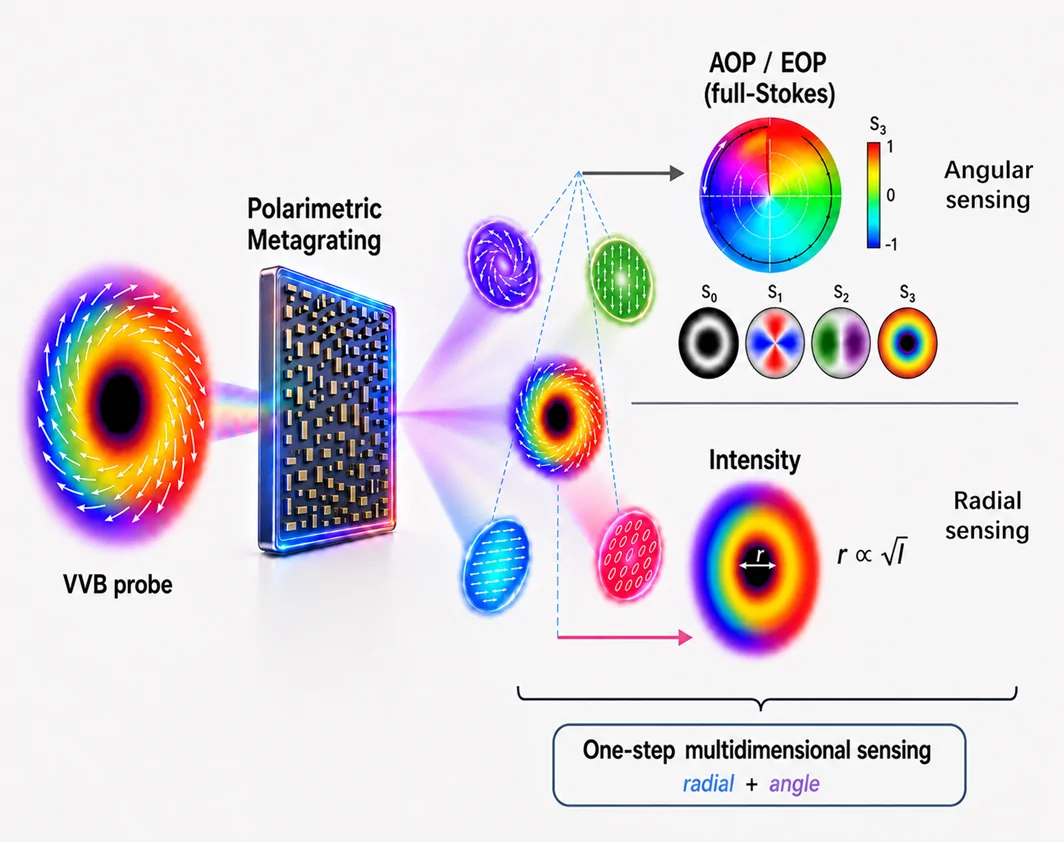

## 1 Introduction

Multidimensional optical sensing [1, 2] has become important in compact photonic metrology [3, 4], laser beam diagnostics [5], optical alignment [6], interface characterization [7], industrial inspection [8], and biomedical imaging [9]. In these scenarios, light intensity or centroid displacement alone is insufficient to characterize the relevant optical response. This limitation calls for a single‐measurement approach capable of retrieving spatial morphology, phase singularity, polarization distribution, angular variation, and mode‐related parameters, thereby providing a more complete description of optical fields and object‐induced perturbations.

Conventional approaches based on interferometric analysis [10], modal decomposition [11, 12], or sequential Stokes polarimetry [13] can provide high sensitivity and rich optical‐field information. However, they commonly rely on bulky optical components, stringent alignment conditions, or multiple measurement steps, limiting their suitability for compact and rapid multidimensional sensing. Structured light [14] provides a powerful platform for addressing these requirements, as its spatial profile, phase structure, and polarization distribution can be flexibly engineered [15, 16, 17]. Among various structured optical fields, optical vortex beams are particularly attractive for multidimensional sensing owing to their helical wavefronts, phase singularities, and well‐defined topological charges [18, 19]. These characteristics provide distinctive spatial signatures for displacement measurement, beam tracking, optical manipulation, and optical mode analysis [18]. However, scalar vortex beams primarily encode information through phase and intensity variations, whereas their spatially uniform polarization leaves the polarization degree of freedom unexploited, thereby constraining the accessible sensing dimensionality.

Vector vortex beams [17, 20, 21] (VVBs) integrate optical vortices with spatially inhomogeneous polarization states [22], introducing an additional degree of freedom for multidimensional optical sensing [21, 23, 24]. In VVBs, the topological charge *l* determines the phase singularity and radial intensity morphology, whereas the polarization order *m* governs the spatial polarization properties. This integration of phase singularity and spatially varying polarization makes VVBs a powerful carrier for multidimensional sensing [25, 26]. Their dark‐core radius, azimuthally varying angle of polarization (AOP), and ellipticity‐related polarization response can provide distinct measurable features associated with radial intensity morphology, azimuthal polarization structure, and polarization‐dependent angular variation. Nevertheless, the respective governing roles of *l* and *m*, as well as their relationships with experimentally measurable VVB characteristics, require systematic investigation. Simultaneous extraction of these features also remains challenging, particularly for elliptically polarized VVBs, whose characterization generally requires full‐Stokes polarimetry implemented through waveplate rotation or multiple sequential measurements. Such procedures reduce system compactness and restrict rapid measurement.

Metasurfaces [27, 28] have emerged as a compact and flexible platform for polarization analysis through the tailored interaction between light and subwavelength nanostructures [29, 30, 31]. Unlike polarization metasurfaces based on spatially interleaved detection regions, polarimetric metagratings [2] diffract the incident light into multiple polarization‐encoded diffraction orders, allowing the full‐Stokes parameters to be reconstructed in a single measurement [32, 33, 34]. Compared with conventional polarimeters based on bulk waveplates and polarizers, polarimetric metagratings offer a chip‐scale platform for analyzing spatially varying vectorial optical fields.

Here, we propose a polarimetric metagrating‐assisted one‐step multidimensional sensing approach using VVBs as optical probes. In this framework, radial features, azimuthal polarization characteristics, and polarization‐dependent angular responses are jointly retrieved within a single measurement. Systematic theoretical and experimental investigations of the topological charge *l* and polarization order *m* reveal their distinct governing roles and lead to a parameter‐space framework linking the intrinsic VVB parameters to measurable optical features. The dark‐core radius extracted from the central diffraction order exhibits a square‐root dependence on the topological charge, with a radius detection resolution of 10 pixels, thereby providing a robust radial sensing feature. Meanwhile, the reconstructed AOP distribution exhibits a 2*m*‐fold periodicity, allowing the azimuthal angular change to be determined from the measured AOP variation and the *m*, thereby introducing a polarization‐resolved angular sensing dimension. The additional use of the central diffraction order for dark‐core radius detection extends the metagrating beyond polarization analysis and broadens its functionality in polarimetric scenarios. The integration of the topological and polarization degrees of freedom of VVBs with chip‐scale full‐Stokes polarimetry establishes a compact and versatile platform for multidimensional optical sensing, including vector‐beam diagnostics, structured‐light state recognition, single‐shot spatial polarimetry, and polarization‐dependent rotation sensing.

## 2 Results and Discussion

### 2.1 Multidimensional‐Sensing Principles Using Vector Vortex Beam

VVBs can be generated through the coherent superposition of two orthogonal vortex modes carrying opposite topological charges in the circular polarization basis, with an appropriate relative phase relationship. Taking the left‐ and right‐handed circular polarization vectors as the basis states, the VVB can be expressed as:

(1) $$\hat{E}=E_{R}{\hat{e}}_{R}+E_{L}{\hat{e}}_{L}=A\left(r_{0}\right)\exp\left(i\delta_{R}\right){\hat{e}}_{R}+A\left(r_{0}\right)\exp\left(i\delta_{L}\right){\hat{e}}_{L},$$

where $\delta_{L}=l_{L}\theta_{0}-\frac{\phi }{2}$ and $\delta_{R}=l_{R}\theta_{0}-\frac{\phi }{2}$ are denoted as the phase topological charges of the left‐ and right‐handed circularly polarized vortex components, respectively. $l_{L}$ and $l_{R}$ represent the phase topological charges of the left‐ and right‐handed circularly polarized vortex components, respectively. $\theta_{0}$ is the azimuthal coordinate and ϕ is the initial phase. $A\;\left(r_{0}\right)$ is the amplitude of the VVB, which is given by:

(2) $$A\left(r_{0}\right)=\left(\frac{\sqrt{2}r_{0}}{w}\right)\exp\left(-\frac{r_{0}^{2}}{w^{2}}\right),$$

where $r_{0}$ is the radial coordinate and w is the beam waist radius. Thereby the field distribution of the VVB at the incident plane can be expressed as:

(3) $${\hat{E}}_{0}\left(r_{0},\theta_{0}\right)=\left(\frac{\sqrt{2}r_{0}}{w}\right)\exp\left(-\frac{r_{0}^{2}}{w^{2}}\right)\left[\begin{array}{l} \cos\left(m\theta_{0}+\frac{\phi }{2}\right)\\ \sin\left(m\theta_{0}+\frac{\phi }{2}\right) \end{array}\right]\exp\left(il\theta_{0}\right),$$

where $m=\frac{l_{L}-l_{R}}{2}$ represents the polarization order of the VVB, and $l=\frac{l_{L}+l_{R}}{2}$ represents the phase topological charge of the VVB.

To investigate the evolution of VVB topological charge *l*, VVBs with *l* ranging from 1 to 10 are numerically simulated by the commercial software MATLAB. It can be seen from Figure 1a, the radius of the central dark‐core region (green circle area) grows with increasing *l.* The dark‐core region starts at the boundary where the amplitude of the VVB equals 1/e^2^ of its peak value. By differentiating Equation (2), the radius of the dark core region corresponding to the peak amplitude can be given by:

(4) $$r_{\text{peak}}=w\sqrt{\frac{\left|l\right|}{2}},$$

Then, by substituting *r*_peak_ into Equation (2), *E*(*r*_peak_) can be written as:

(5) $$\left|E\left(r_{\text{peak}}\right)\right|={\left(\sqrt{\left|l\right|}\right)}^{\left|l\right|}\cdot e^{-\left|l\right|/2}={\left|l\right|}^{\left|l\right|/2}e^{-\left|l\right|/2},$$

Thus, *E*(*r*_dark_) is calculated by:

(6) $$\left|E\left(r_{\text{dark}}\right)\right|=\frac{1}{e^{2}}\left|E\left(r_{\text{peak}}\right)\right|,$$

Consequently, *r*_dark_ is expressed as:

(7) $$r_{\text{dark}}\approx \frac{1}{\sqrt{2}}w\left(z\right)\sqrt{\left|l\right|}\propto \sqrt{\left|l\right|}.$$

**FIGURE 1:**
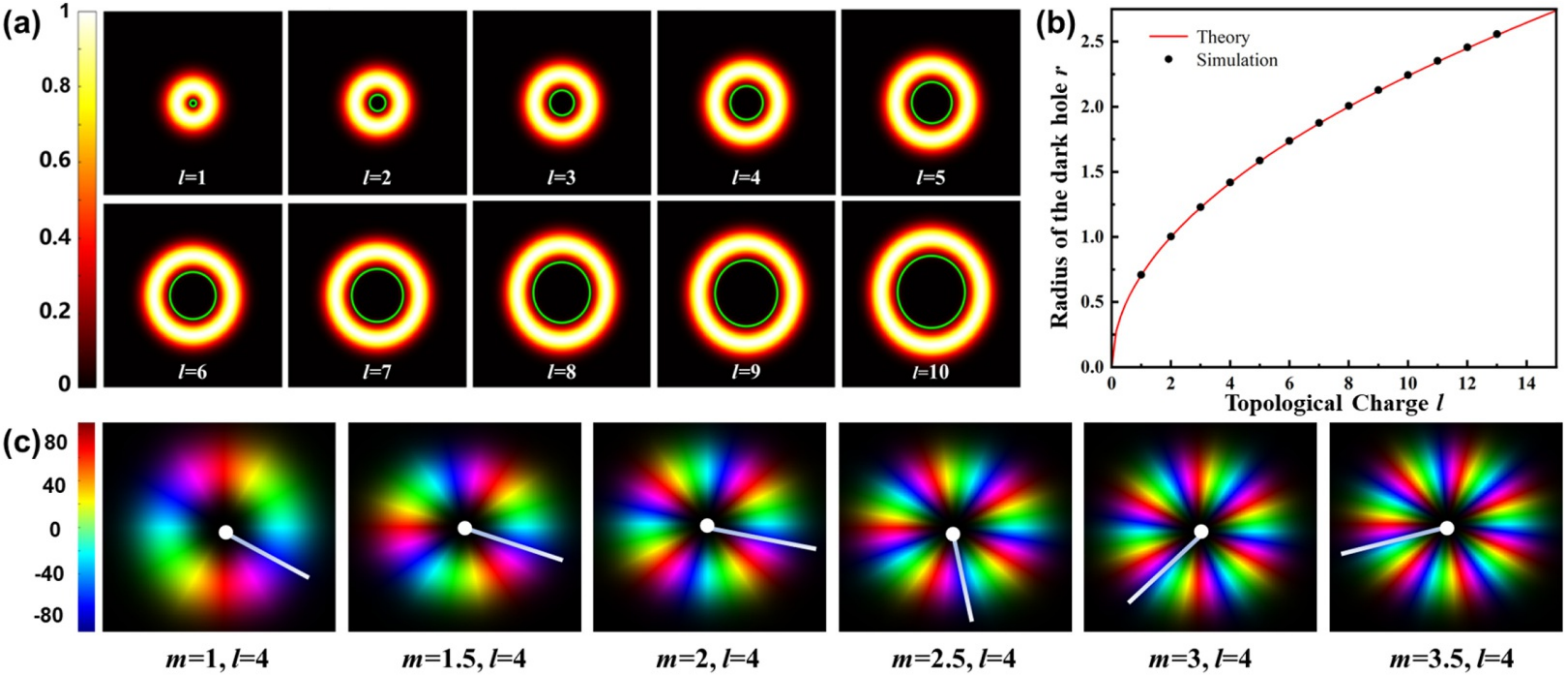
(a) Simulated intensity distributions of VVBs with *m* = 1 for *l* = 1 to 10. Green circle indicates the region of the dark core. (b) Dark‐core radius versus topological charge, exhibiting a square‐root dependence. (c) AOP distributions of the VVBs with *l* = 4 for *m* = 1, 1.5, 2, 2.5, 3 and 3.5.

The simulated radius of the dark‐core region is measured and plotted as a function of the *l*. As shown in Figure 1b, the dark‐core radius of the VVB exhibits a square‐root dependence on the topological charge, consistent with the theoretical derivation in Equation (7). This relationship quantitatively correlates the topological charge to the dark‐core radius, establishing a radial sensing feature within the multidimensional framework.

Thereafter, we study how the AOP distribution varies with the polarization order *m* of the VVB. As illustrated in Figure 1c, with *l* fixed, the number of AOP periods over the full 360° azimuthal range increases with *m* and is equal to 2*m*. Since the AOP is defined within 0°–180°, the azimuthal positions corresponding to a given AOP value scale inversely with *m*, and the angular separation between neighboring azimuthal positions is given by 180°/m, as marked by the white lines. According to Equation (3), the value of AOP can be written as:

(8) $$\text{AOP}=m\theta +\frac{\phi }{2}.$$

On this basis, the relationship between the changes in AOP and the spatial azimuthal angle can be calculated by:

(9) ΔAOP=mΔθ.

In conclusion, the phase singularity and spatially varying AOP distribution of VVBs are characterized by the topological charge *l* and polarization order *m*, which govern the dark‐core radius and AOP periodicity, respectively. This correspondence establishes an (*l*, *m*)‐based framework for multidimensional optical sensing.

### 2.2 Experimental Implementation

#### 2.2.1 The Generation of Vectorial Vortex Beam

The optical setup is designed to generate two orthogonal circularly polarized vortices to produce VVB, as indicated by the green dotted box in Figure 2a. First, the expanded 488 nm laser beam passes through a half‐wave plate (HWP) and a quarter‐wave plate 1 (QWP1) to become a right circularly polarized light (RCP), and then is modulated to be a left circularly polarized (LCP) vortex beam with a topological charge of *l*_1_ by the vortex retarder (VR). Subsequently, quarter‐wave plate 2 (QWP2) converts the beam into linearly polarized light oriented at 45° relative to the principal axis (*x*‐axis in the figure) of the spatial light modulator (SLM). The beam is then split into Beam 1 and Beam 2 by the non‐polarizing beam splitter. Beam 1 is directed onto the SLM, which is loaded with a vortex phase of topological charge *l*_2_. Because the SLM modulates only the polarization component parallel to its principal axis, the reflected beam is *x*‐polarized and therefore carries a total topological charge of *l*_1_ + *l*_2_. Beam 2 remains *y*‐polarized and retains the topological charge *l*_1_. The recombined output beam thus is accordingly described as a superposition of an *x*‐polarized vortex beam with charge *l*_1_ + *l*_2_ and a *y*‐polarized vortex beam with charge *l*_1_. After passing through quarter‐wave plate 3 (QWP3), these orthogonal linear polarization states are converted into circular polarization states, yielding an LCP component with topological charge *l*_1_ and an RCP component with topological charge *l*_1_ + *l*_2_, thereby generating the VVB. The Jones Matrix of the beam can be written as:

(10) $$J_{l,m}=e^{il\varphi }\left(\begin{array}{l} \cos\left(m\varphi +\varphi_{0}\right)\\ \sin\left(m\varphi +\varphi_{0}\right) \end{array}\right)=\frac{1}{2}e^{i\left(\left(l+m\right)\varphi +\varphi_{0}\right)}\left(\begin{array}{l} 1\\ -i \end{array}\right)+\frac{1}{2}e^{i\left(\left(l-m\right)\varphi +\varphi_{0}\right)}\left(\begin{array}{l} 1\\ i \end{array}\right),$$

where $\left|l\right|=\frac{2l_{1}+l_{2}}{2}$ is the order of the phase singularity, representing the total quantum number of the beam. $m=\frac{l_{2}}{2}$ is the polarization order. Notably, the VR operates as a spatially variant wave retarder whose local optical axis varies with the azimuthal angle, such that each point on the cross‐section is equivalent to a local HWP plate. In this configuration, the VR controls the topological charge of the output VVB, whereas the SLM modulates the polarization order.

**FIGURE 2:**
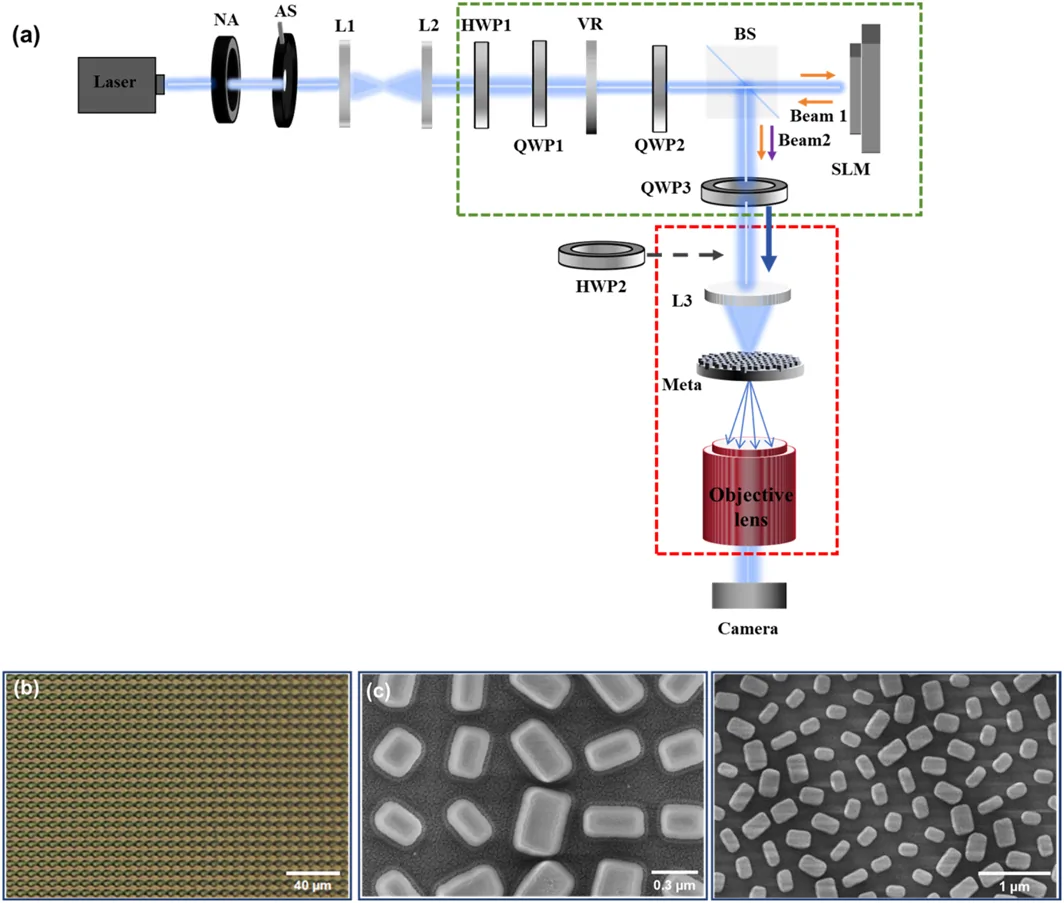
(a) Experimental setup for VVB generation and its polarization analysis. HWP, half‐wave plate; L_1_, L_2_, and L_3_, lens with focus length of 40, 100, and 30 mm; Meta, polarimetric metagrating; ND, neutral attenuator; QWP, quarter‐wave plate; SLM, spatial light modulator; VR, vortex retarder. (b) Optical image of the fabricated metagrating sample captured by a 5× microscope. (c) Scanning electron microscope (SEM) images of the fabricated metagrating sample.

#### 2.2.2 Polarization Analysis by the Polarimetric Metagrating

The polarimetric metagrating, a novel metasurface‐based polarimetry device, enables one‐step full‐Stokes polarization analysis by diffracting the incident light into multiple orders carrying encoded polarization information. This capability allows one‐step quantitative characterization of the polarization states of vector vortex beams (VVBs), particularly elliptical VVBs. Furthermore, the polarimetric metagrating possesses a central diffraction order free of polarization information, which can clearly reveal the VVB pattern, making it highly suitable for studying the relationship between topological charge and radius. In this paper, as shown in Figure 2b, we design a 1 mm × 1 mm polarimetric metagrating with four diffraction orders (Details are provided in the Supporting Information S1: Section S2) for VVB polarization analysis.

The polarization analysis module consists of a polarimetric metagrating and a lens (red dotted box in Figure 2a), where the lens is used to reduce the beam size ensuring entire VVB is processed by the metasurface. Finally, the polarization‐resolved patterns are magnified by a 10× objective lens and recorded with a CMOS camera.

The polarization state of the VVB can be obtained by the equation:

(11) $$AS_{\text{in}}=I,$$

where S_in_ refers to the Stokes vector of the VVB incident on the metagrating, which represents the polarization state to be analyzed. *I* is a 4 × 1 column vector referring to the intensities of the four diffraction orders. *A* is a 4 × 4 instrument matrix, with each row representing one of the four polarization diffraction orders of the metagrating. Therefore, the Stokes vector of VVB can be determined from *A* and the calculated intensities from the camera. *A* is measured by the instrument calibration process (Details are provided in the Supporting Information, Section 2.3). After the calibration, the phase information is loaded on the SLM and the VRs with different topological charges are inserted into the optical path.

### 2.3 Experiment Results

The respective roles of the topological charge and polarization order to radial and angular feature encoding are experimentally investigated and verified. To validate the correlation between the topological charge and the dark‐core radius, VVBs of *m* = 2, *l* = 3, 4, and 6 are first generated by employing VRs with *l*_1_ = 1, 2, and 4, together with the SLM loaded with a topological charge *l*_2_ = 4. As shown in Figure 3, the dark‐core radius exhibits an increasing trend as the topological charge increases, in agreement with the theoretical simulations. Since no polarization information is encoded in the central diffraction order of the metagrating, the dark‐core radius can be clearly identified, as indicated by the red circle in Figure 3b. Using a CMOS camera calibrated at 20 μm per pixel, the dark‐core radii are measured as 37, 47, and 82 pixels, respectively, confirming their square‐root dependence on the topological charge *l* and demonstrating a minimum radial difference of 10 pixels (200 μm).

**FIGURE 3:**
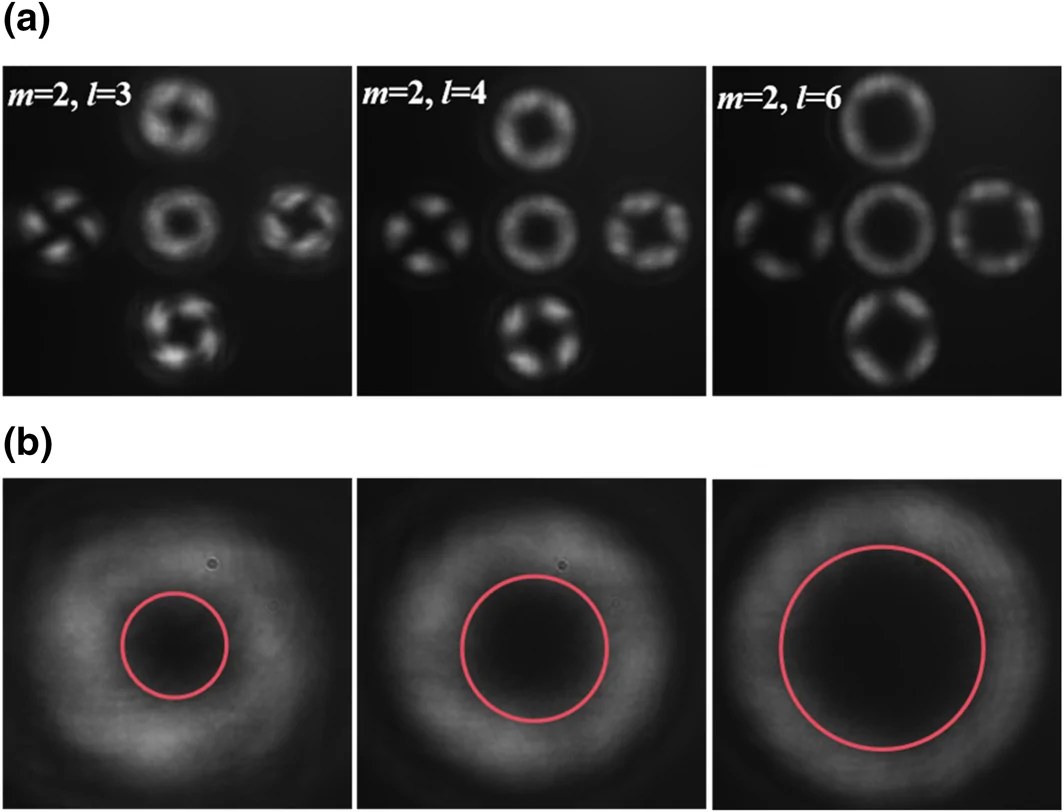
(a) Polarization‐resolved intensity images of different VVBs for fixed *m* and varying *l* at the four metagrating diffraction orders. (b) The corresponding enlarged views of the dark‐core radii in the central diffraction order.

Due to the limited availability of VRs, the topological charge of the generated VVBs could not be kept fixed as *m* changed during the verification of the AOP distribution. We therefore first demonstrate that the polarization characteristics of VVBs are independent of *l*. As shown in Supporting Information S1: Figure S3, both simulation and experiment confirm that the AOP distributions of VVBs remain unchanged as *l* varies. During the experiment, a VR with *l*_1_ = 2 is kept fixed, and the SLM is sequentially loaded with topological charges of *l*_2_ = 2,3,4,5,6, and 8, producing VVBs with (*m*, *l*) = (1,3) (1.5, 3.5) (2, 4) (2.5, 4.5) (3, 5) and (4, 6). As shown in Figure 4a, the polarization‐resolved intensity images at the four diffraction orders of the metagrating exhibit flower‐like patterns, with the number of petals equal to 2*m*. After the four images are aligned and analyzed, the resulting AOP distribution in Figure 4b reveals a periodicity of 2*m* for each VVB, consistent with the number of petals and in agreement with the simulation results. As shown in Supporting Information S1: Figure S4, simulations of the experimentally generated VVBs further confirm that the AOP distribution is governed solely by *m*, whereas the periodic shift in the azimuthal positions corresponding to a given AOP can also be observed.

**FIGURE 4:**
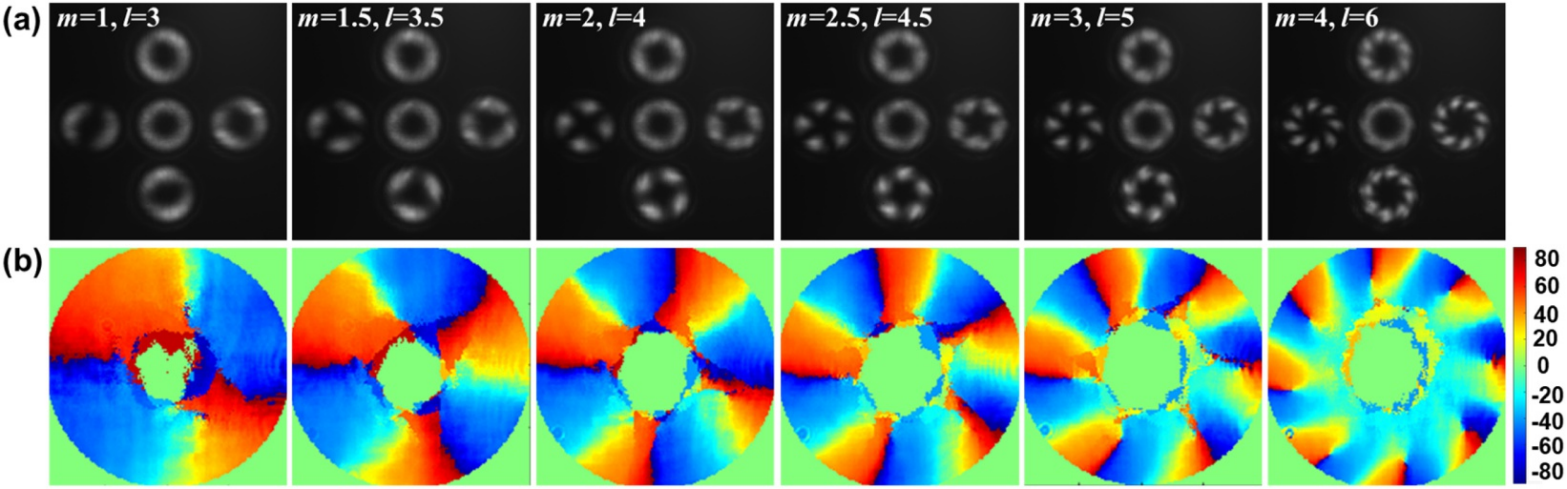
(a) Polarization‐resolved intensity images of different VVBs at the four metagrating diffraction orders. (b) The correspondence AOP distributions.

To further validate the effectiveness and applicability of polarization‐distribution features in characterizing angular variations, we first investigate linearly polarized VVBs whose helical phase structures are identical; their polarization states vary spatially in linear form. According to the formation principle of VVBs, linearly polarized VVBs can be experimentally generated by rotating HWP1 in Figure 2a. Using this configuration, we produce VVBs with *m* = 2 and *l* = 4, for which the polarization components are given by $\left[\begin{array}{l} \cos\left(m\theta \right)\\ -\sin\left(m\theta \right) \end{array}\right]$. When linearly polarized VVBs are used as probes, an HWP is placed behind QWP3 as the corresponding target objects, and the angular change is obtained from the AOP. The angular position of five randomly selected points on the HWP are tracked as it is rotated in 2° increments.

As seen in Figure 5a, although the variation under a 2° rotation is not readily discernible for linearly polarized VVBs, after a 10° rotation, the AOP values of the original five points change obviously, indicating that the angular change can be inferred from the variation of AOP. The AOP changes at each point are summarized in Table 1. Since both the insertion and rotation of the HWP affect the AOP, the intrinsic polarization transformation introduced by the HWP should be calibrated out before quantifying the relationship between the rotation angle and AOP variation. After subtracting the 4° AOP change induced by a 2° HWP rotation, Figure 5b reveals a linear relationship between the angular change and the calibrated AOP variation, consistent with Equation (9) (Details are provided in the Supporting Information S1: Section S4).

**FIGURE 5:**
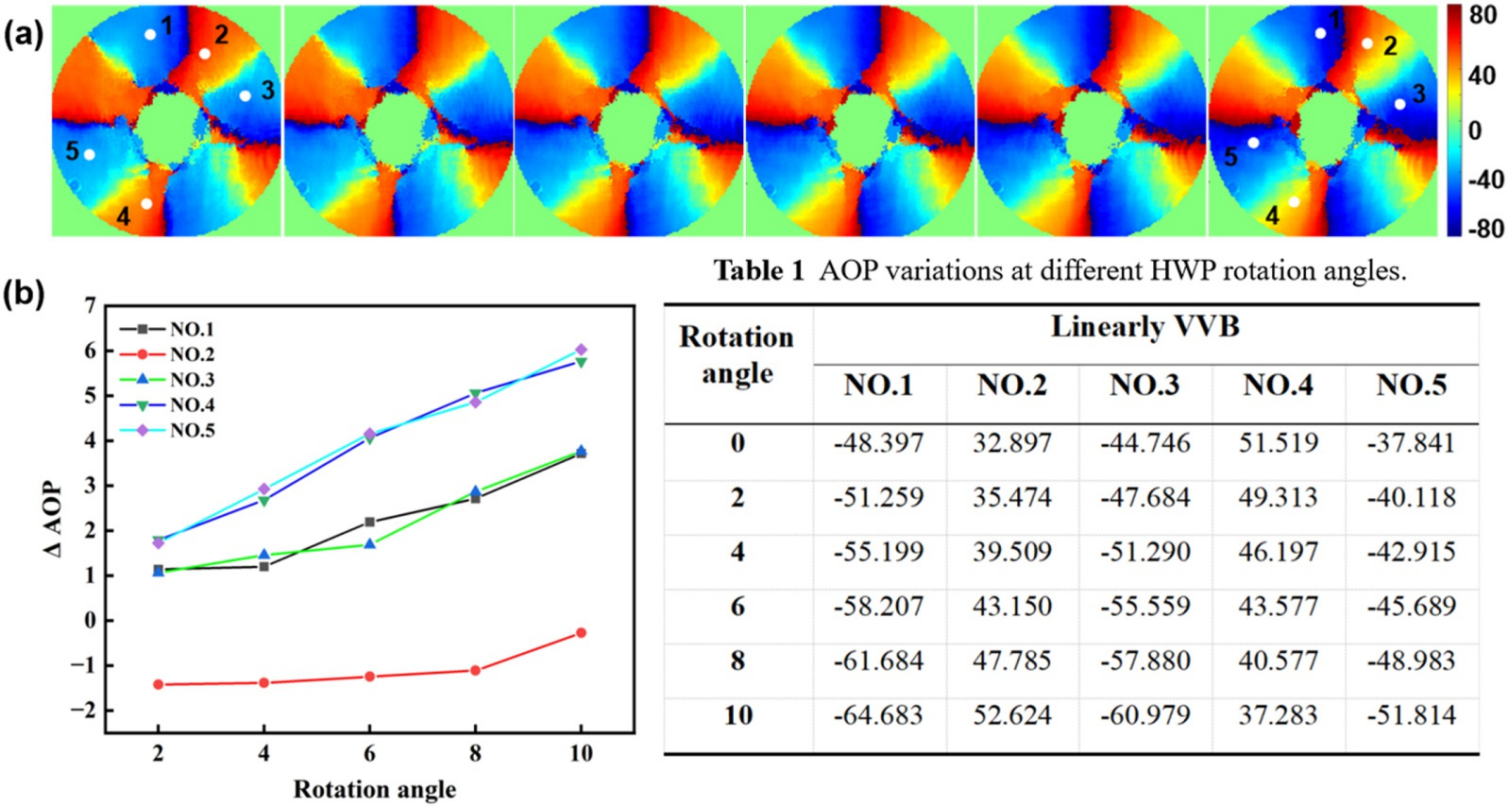
(a) Schematic illustration of the angular changes of five randomly selected points using the AOP variation of a linearly polarized VVB. (b) Angle of polarization (AOP) versus half‐wave plate rotation angle. Table 1: Quantified AOP values of randomly selected points under half‐wave rotation angles.

**TABLE 1:** AOP variations at different HWP rotation angles.

Rotation angle	Linearly VVB				
	NO.1	NO.2	NO.3	NO.4	NO.5
0	−48.397	32.897	−44.746	51.519	−37.841
2	−51.259	35.474	−47.684	49.313	−40.118
4	−55.199	39.509	−51.290	46.197	−42.915
6	−58.207	43.150	−55.559	43.577	−45.689
8	−61.684	47.785	−57.880	40.577	−48.983
10	−64.683	52.624	−60.979	37.283	−51.814

We also conduct additional experiments using elliptically polarized VVBs characterized by *m* = 2 and *l* = 4, whose polarization components are expressed as $\left[\begin{array}{l} \cos\;\alpha \cdot e^{\text{im}\theta }+\sin\;\alpha \cdot e^{-\text{im}\theta }\\ i\left(\cos\;\alpha \cdot e^{\text{im}\theta }-\sin\;\alpha \cdot e^{-\text{im}\theta }\right) \end{array}\right]$. In contrast to linearly polarized VVBs, they exhibit spatially varying elliptical polarization states. The generation of polarized VVBs is realized by rotating QWP1 in Figure 2a. An additional QWP is therefore placed after QWP3 and used as the target project, with the angular variation retrieved from the corresponding ellipticity angle of polarization (EOP) response. The EOP values at five randomly selected spatial points are recorded as the QWP is rotated in 2° increments. Although successive variations remain subtle, a clear EOP response emerges when the accumulated rotation reaches 10°, demonstrating that elliptically polarized VVBs can also support angular sensing through their EOP response (Details are provided in the Supporting Information S1: Section S4).

The experimental results demonstrate a minimum distinguishable rotation step of 2° for both linear and elliptical VVBs, indicating that the proposed approach supports angular sensing across the full‐Stokes space.

## 3 Conclusion

In conclusion, we have developed a polarimetric metagrating‐assisted one‐step multidimensional sensing approach using VVBs as information carriers. Through systematic theoretical and experimental investigations of the topological charge *l* and polarization order *m*, we elucidate their distinct governing roles and establish a parameter‐space framework correlating the intrinsic beam parameters to radial, angular, and polarization‐resolved sensing features. Measurements with linearly and elliptically polarized VVBs further demonstrate polarization‐dependent angular sensing, with rotational increments of approximately 2° experimentally distinguishable. Supported by the single‐step full‐Stokes capability of the polarimetric metagrating, the proposed approach establishes a compact and efficient multidimensional sensing framework that extends vortex‐beam‐based sensing beyond conventional phase‐ and intensity‐dependent information, with potential applications in beam alignment and stability monitoring, structured‐light state recognition, and polarization metrology. In addition, the effective use of the polarization‐insensitive central diffraction order of the polarimetric metagrating broadens its application scope beyond polarimetry.

## Author Contributions

**Han Gao:** conceptualization, methodology, funding acquisition, writing – original draft, formal analysis. **Zhelei Chen:** methodology, formal analysis, validation, data curation. **Kaihao Deng:** methodology, investigation. **MinJun Xie:** methodology, investigation. **Huanxin Liao:** investigation. **Zeyu Zheng:** methodology. **Hao Yang:** investigation. **ChunLian Zhan:** supervision. **Yanlong Meng:** resources. **Bo Xiong:** conceptualization, funding acquisition, writing – review and editing. **Wei Ma:** conceptualization, funding acquisition, writing – review and editing.

## Conflicts of Interest

The authors declare no conflicts of interest.

## Supporting information


Supporting Information S1

## Data Availability

The data that support the findings of this study are available from the corresponding author upon reasonable request.
