# Supplementary material for: Polarimetric Metagrating‐Assisted One‐Step Multi‐Dimensional Sensing Using a Vector Vortex Beam
Source: Nanophotonics. 2026 Aug 24;15(16):e70263. doi: 10.1002/nap2.70263 (PMC13672163; doi:10.1002/nap2.70263)
Supplement: Supplementary file 1 [file NAP2-15-e70263-s001.docx]

**Supporting Information for**

**Polarimetric metagrating-assisted one-step multi-dimensional sensing using a vector vortex beam**

Han Gao^1,3,*^, Zhelei Chen^1^, Kaihao Deng^2^, MinJun Xie^2^, Huanxin Liao^1^, , Zeyu Zheng^1^, ChunLian Zhan^1,3^, Yanlong Meng^1,3^, Bo Xiong^2,*^, Wei Ma^2,*^

^1^China Jiliang University, College of Optical and Electronic Technology, Hangzhou, 310018, China.

^2^College of Information Science and Electronic Engineering, Zhejiang University, Hangzhou, 310027, China.

^3^Zhejiang Province Key Laboratory of Optoelectronic Metrology and Precision Instruments, Hangzhou, 310018, China.

*Corresponding authors:

gaohan@cjlu.edu.cn, [ma_wei@zju.edu.cn](mailto:ma_wei@zju.edu.cn), [bo.xiong@zju.edu.cn](mailto:bo.xiong@zju.edu.cn)

**1. Vector vortex beam numerical simulation**

Numerical simulations of VVB generation and polarization analysis are conducted by the commercial software MATLAB. The incident light is set to a wavelength of 488 nm with a beam waist of 1 μm. VVBs with different topological charges are generated through the coherent superposition of left- and right-circularly polarized light. As shown in Figure S1, the numerically simulated VVBs with *m*=1 and *l*=2, 5 exhibit distinct phase distributions and polarization-resolved intensity patterns, confirming the effectiveness of the simulation.

Figure S1. Simulated phase distribution of VVBs with *m*=1, *l*=2 (a) and *m*=1, *l*=5 (b). Simulated 0° polarization-resolved intensity distribution of VVBs with *m*=1, *l*=2 (c) and *m*=1, *l*=5 (d). Simulated 90° polarization-resolved intensity distribution of VVBs with *m*=1, *l*=2 (e) and *m*=1, *l*=5 (f).

**2. Design and preparation of the polarimetric metagrating**

**2.1 Polarimetric metagrating design**

The design of the polarimetric metagrating is based on previous reported method^1^. Since the diameter of the dark core of the VVB increases with the topological charge, the metagrating size was set to 1 mm ×1 mm to ensure effective analysis of the polarization state of the VVB.

Simulations of the transmission and phase distributions of the metasurface were done by FDTD. The height of the nanopillar is fixed at 750 nm, with a unit cell size of 450 nm × 450 nm. The nanofin diameters *W_x_* and *W_y_* varies from 100 nm to 350 nm. For each pillar, a far-field monitor is placed a few wavelengths above the structure. The transmission $\left\{ t_{x} \right.\left. {,t}_{y} \right\}$and phase $\left\{ \phi_{x} \right.\left. {,\phi}_{y} \right\}$properties of the silicon nitride nanocuboids for the 15876 elements are stored as a (MATLAB) library. The phase optimization is performed by “*fun*” function in Python. Python’s inbuilt ***patternsearch*** is employed to perform the global search. During the search process, for each phase profile iteration, the Fourier coefficients and the figure of merit are calculated by the metasurface phase and transmission profiles from the metasurface library.

Figure S2. Simulated phase shift $\phi_{x}$ for x-polarized light (a) and $\phi_{y}$ for y-polarized light (b)

a function of the length (*W_y_*) and width (*W_x_*) of the nanocuoboids at the wavelength of 488 nm. Simulated transmission amplitude $\left| t_{x} \right|^{2}$ for x-polarized (c) $\left| t_{y} \right|^{2}$y-polarized (d) light as a function of the length (*W_y_*) and width (*W_x_*) of the nanocuboids at the wavelength of 488 nm. Simulated generated polarization states in four diffraction orders at 488 nm (e). The solid line represents FDTD simulated results. Dashed line represents theoretical polarization state.

**Polarimetric Metagrating Parameters**

|  | Phase | | Geometry | | Rotation angle |
| --- | --- | --- | --- | --- | --- |
| Element |  |  | $w_{x}$ | $w_{y}$ | θ |
| 1 | 0.43 | 2.35 | 160 | 300 | -2.35 |
| 2 | -1.28 | 0.4 | 134 | 218 | 2.67 |
| 3 | 0.78 | -1.53 | 254 | 124 | -1.71 |
| 4 | -2.75 | 1.04 | 350 | 172 | 0.01 |
| 5 | 2.3 | 1.04 | 280 | 180 | 2.77 |
| 6 | 2.05 | -2.86 | 202 | 336 | -0.37 |
| 7 | -0.77 | -2.74 | 206 | 102 | -0.8 |
| 8 | 0.01 | -2.74 | 232 | 102 | 2.04 |
| 9 | -3.07 | 0.99 | 346 | 170 | -2.8 |
| 10 | 1.76 | 2.82 | 202 | 300 | 2.06 |
| 11 | 1.58 | -2.33 | 190 | 350 | -0.15 |
| 12 | -0.56 | -2.2 | 208 | 108 | -0.33 |
| 13 | -2.71 | -0.79 | 102 | 204 | -2.35 |
| 14 | -2.98 | 0.21 | 350 | 152 | 0.63 |
| 15 | 1.99 | -2.74 | 198 | 346 | -0.43 |
| 16 | -2.29 | 0.57 | 102 | 280 | 1.72 |
| 17 | -0.23 | -1.85 | 208 | 118 | 0.16 |
| 18 | -1.8 | -0.94 | 124 | 180 | -1.95 |
| 19 | -2.69 | -0.81 | 102 | 204 | -2.36 |
| 20 | 1.61 | -2.43 | 190 | 350 | 3.04 |
| 21 | 0.2 | 3.11 | 150 | 350 | -2.57 |
| 22 | -0.04 | -1.69 | 214 | 120 | 0.59 |
| 23 | -1.12 | 0.37 | 138 | 214 | 2.74 |
| 24 | 0.83 | -1.69 | 284 | 110 | 1.39 |
| 25 | 2.33 | 0.43 | 300 | 160 | -0.78 |

Table S1. Optimized phase and geometry parameters of the polarimetric metagraing.

**2.2 Preparation of the polarimetric metagrating**

A 750 nm SiN layer was deposited onto a 500 μm quartz glass substrate by using low pressure chemical vapor deposition (LPCVD). After cleaning the sample, poly (methyl methacrylate) (PMMA) was spin-coated onto the silicon layer and baked at 180℃ for 120s. To prevent charge accumulation, a conductive polymer was spin-coated onto the PMMA. Then the resist pattern was exposed by electron beam lithography, followed by developing in an ethyl acetate solution. Subsequently, the sample was immersed in a fixing solution to remove the developer. A 60 nm chromium (Cr) was deposited onto the sample as a hard mask via electron beam evaporation, while the remained PMMA regions were lift-off in an acetone solution. After that, the Cr hard mask was transferred to the silicon layer, and the 750 nm SiN layer was etched by an inductively coupled plasma etching system. The fabricated metasurface grating sample is characterized by the optical microscope (BH200MRT 3000X, SUNNY GROUP CO., LTD) and a cold-field emission scanning electron microscope (S-4800, HITACHI). The total area of the metasurface grating is 1 mm ×1 mm.

**2.3 Instrument matrix calibration**

Because the metasurface grating deviates from ideal performance under the influence of multiple factors, instrument calibration is required before polarimetric measurement to determine the instrument matrix ***A***. The calibration process is conducted following the reported work^2^. In our experiment the instrument process is done based on the optical setup in Figure 2(a) without inserting VR and loading phase information on the SLM. For linear polarization, the half-wave plate (HWP) is rotated from 0° to 180° in steps of 5° to obtain 19 sets of intensity images. By fitting the variation of the intensity ***I*** with the incident polarization angle θ for each polarization analysis channel, the first three column of the instrument matrix are thereby calibrated. For circular polarization calibration, the QWP and HWP are rotated to generate 5 sets of let- and right- circularly polarized light, and the corresponding intensity are measured and averaged. The last column of the instrument matrix is then calibrated, yielding the final instrument matrix.

**3. Optical Setup**

A 488 nm continuous-wave laser (PSL22-488-20mW, LBTEK) is used as the incident light source, and a quarter-/half-wave plate combination is employed to generate circularly polarized light. Vortex retarders (VR1-488, LBTEK) with orders *l*_1_=1, 2 and 4 are used to control the topological charges of the VVBs. A spatial light modulator (SLM; FSLM-2k73-P02HR, CAS Microstar) is used to modulate the polarization orders *m* of the VVBs. A 10× apochromatic objective lens (MY10X-823, Mitutoyo) is applied to obtain clear images at the four diffraction orders. The images are recorded by the CMOS camera (C11440-36U, HAMAMATSU).

**4.** **Vector vortex beam multidimensional sensing supplement experiment result**

Figure S3. Experiment (a) and Simulation (b) AOP distribution of VVBs with *m*=2, *l*= 3, 4 and 6

Figure S4. Simulation AOP distributions of the same VVBs used in the experiment.

The Jones vector of linearly polarized light and the Jones matrix of the HWP are respectively expressed as $\widehat{E}=E_{0}\left(\begin{matrix} \cos\theta_{\text{in}} \\ \sin\theta_{\text{in}} \end{matrix}\right)$ and. The resulting AOP change upon insertion of the HWP is then given by：

.

Consequently, a 2° rotation of the HWP induces a 4°change in the AOP. The Jones vector of the incident elliptically polarized light is given by $\widehat{E}=E_{0}\left(\begin{matrix} \cos\theta_{\text{in}} \\ \sin\theta_{\text{in}}e^{-i\delta} \end{matrix}\right)$, and the Jones matrix of the QWP is expressed as. The QWP-induced variation in the EOP can then be derived as ：

This relation shows that the EOP is locally linear within each monotonic interval but exhibits an overall periodic, piecewise-linear response. Therefore, its variation cannot be described by a single proportionality factor with the QWP rotation angle. Although a direct analytical relationship between the rotation angle and EOP variation cannot be established, the experimental results reveal an approximately linear dependence, as summarized in Table S1 and illustrated in Figure S5.

Figure S5. (a) Schematic illustration of the angular changes of five randomly selected points using the EOP variation of an elliptically polarized VVB. (b) Ellipticity angle of polarization (EOP) versus quarter-wave plate rotation angle. Table S1: Quantified EOP values of randomly selected points under different.

Figure S6. The AOP (a) EOP (b) distributions of experimentally generated linearly, circularly and elliptically polarized VVBs.

References

1	Rubin, N. A. *et al.* Imaging polarimetry through metasurface polarization gratings. *Optics Express* **30**, 9389-9412 (2022).

2	Rubin, N. A. *et al.* Polarization state generation and measurement with a single metasurface. *Optics express* **26**, 21455-21478 (2018).
